# Periodontal Treatment Improves Serum Levels of Leptin, Adiponectin, and C-Reactive Protein in Thai Patients with Overweight or Obesity

**DOI:** 10.1155/2021/6660097

**Published:** 2021-02-02

**Authors:** Notkamon Wanichkittikul, Penpan Laohapand, Chayasin Mansa-nguan, Supanee Thanakun

**Affiliations:** ^1^Department of Oral Medicine and Periodontology, Faculty of Dentistry, Mahidol University, Bangkok, Thailand; ^2^Department of Clinical Tropical Medicine, Faculty of Tropical Medicine, Mahidol University, Bangkok, Thailand; ^3^College of Dental Medicine, Rangsit University, Pathumthani, Lak Hok, Thailand

## Abstract

Periodontitis and overweight or obesity independently change serum levels of leptin, adiponectin, and C-reactive protein (CRP). The aim of this study is to investigate the alterations of serum levels of leptin, adiponectin, and CRP after nonsurgical periodontal treatment (NSPT) in Thai patients with overweight or obesity (Owt/Ob) who did or did not exhibit severe periodontitis (SP) and normal weight (Nwt) patients with or without SP. Two hundred sixty patients were screened; 29 patients were included in this study. The study participants comprised 6 patients with Owt/Ob who exhibited SP, 11 patients with Owt/Ob who did not exhibit SP, 5 Nwt patients with SP, and 7 Nwt patients without SP. Periodontal disease status was evaluated; serum levels of leptin, adiponectin, and CRP were determined by enzyme-linked immunosorbent assay at baseline, as well as at 3 and 6 months after NSPT. At 3 months after NSPT, periodontal status was improved in all groups (*p* < 0.05), except Nwt patients without SP. Serum levels of leptin and CRP were significantly reduced, while serum levels of adiponectin were elevated after NSPT, regardless of bodyweight or waist circumference (*p* < 0.05). Improvement in serum levels of leptin after NSPT was also observed in the Nwt with SP group (*p* = 0.015); these levels did not significantly differ in Nwt patients without SP. NSPT reduces serum levels of leptin and CRP and enhances serum levels of adiponectin in Thai patients with Owt/Ob, irrespective of periodontitis severity. These results suggest a role for periodontal treatment in the systemic inflammatory response of Thai people with Owt/Ob.

## 1. Introduction

Overweight (Owt) and obesity (Ob) are highly prevalent chronic diseases in Thailand and worldwide [1, 2]. During the development of obesity, adipocytes dysregulate adipokine production, which contributes to local and systemic inflammation and disturbances in glucose homeostasis [3], and these These adipokines include proinflammatory cytokines (e.g., leptin, tumor necrosis factor-*α*, and interleukin-6) and anti-inflammatory cytokines (e.g., adiponectin), all of which have multiple metabolic balance functions [3, 4]. Furthermore, delivery of increased levels of the tumor necrosis factor-*α* and interleukin-6 to the liver may cause liver inflammation and contribute to elevated production of C-reactive protein (CRP) [5]. Therefore, Ob, associated with a chronic low-grade systemic inflammatory state, is considered a predisposing factor for a variety of diseases, including cardiovascular disease and type 2 diabetes mellitus [1, 3].

In addition to its presence in people with Owt or Ob, chronic inflammation in the body may be found in patients with periodontitis, a major source of oral inflammation [3]. Many previous studies have shown elevated levels of serum inflammatory mediators such as CRP, the tumor necrosis factor-*α*, and interleukin-6 in patients with periodontitis; these are similar to the inflammatory mediators found in patients with Owt and Ob [3, 6, 7].

An association between Ob and periodontitis was previously reported [8–10]. Variation in the production of local and systemic inflammatory mediators was related to BMI and periodontal disease [11–13]. Thus far, the mechanism explaining the relationship between Owt or Ob and periodontitis is not fully understood. Inflammatory mediators secreted by either of the two diseases may constitute a bidirectional link [3]. Thanakun and Izumi demonstrated a significant elevation of plasma CRP, but a reduction of adiponectin, in patients with severe periodontitis (SP), irrespective of body mass index (BMI) [14]. Patients with Owt or Ob had higher plasma levels of CRP and leptin, but lower levels of adiponectin, compared with normal weight (Nwt) participants, irrespective of periodontitis status [14]. Several studies of serum cytokine levels before and after periodontal therapy have been performed, along with analyses of clinical changes [15–20]. Cytokines often assessed in these studies include CRP, leptin, adiponectin, interleukin-1*β*, interleukin-6, and the tumor necrosis factor-*α* [16–20]. However, the results have been inconsistent among studies and have only been explored in two groups of patients: people with Owt or Ob who exhibit periodontitis, compared with Nwt people who exhibit periodontitis [15–18, 20]. Moreover, the study previously performed by our group was a cross-sectional investigation [14]. A role for periodontal treatment in the decrease of systemic inflammatory response was anticipated. Therefore, a prospective interventional study in patients with Owt or Ob who do or do not exhibit SP, compared with Nwt individuals with or without SP, is needed. The objective of the present study was to investigate changes in serum leptin, adiponectin, and CRP levels after nonsurgical periodontal treatment (NSPT) in Thai patients with Owt or Ob who did or did not exhibit SP, compared with Nwt patients with or without SP. We hypothesized that NSPT would improve serum levels of these inflammatory mediators in patients with Owt or Ob.

## 2. Materials and Methods

### 2.1. Ethics Approval and Consent

This observational study was approved by the Institutional Review Board for human subjects of Mahidol University (COA. No. MU-DT/PY-IRB 2016/060.0411) and conducted in full accordance with the Declaration of Helsinki. The study was registered in the Thai Clinical Trials Registry on the WHO International Clinical Trial Registry Platform (registration number: TCTR20170607003). All participants received information regarding the study protocol and provided written consent to participate.

### 2.2. Participants and Assessment of Owt or Ob

Patients who attended the General and Special Clinic, Hospital for Tropical Diseases, Faculty of Tropical Medicine, Mahidol University, from November 2016 to December 2018 were recruited for this study. Inclusion criteria were as follows: Thai ethnicity, age ≥35 years, and possession of ≥9 teeth (excluding third molars and teeth indicated for extraction). Demographic data, medical histories, and dental histories of potential participants were collected by interview. Their levels of triglycerides, total cholesterol, and fasting blood sugar (FBS) within the preceding 6 months were evaluated from medical records. Potential participants were excluded if they met any of the following criteria: (1) presence of systemic diseases/conditions related to periodontitis and/or Ob, such as diabetes mellitus (FBS ≥ 126 mg/dl), hypertension, or cardiovascular disease; (2) presence of diseases that affect periodontal disease progression or treatment and/or gain/loss of weight, such as immunological disorders or osteoporosis; (3) current smoking habit or smoking habit within the past 1 year; (4) pregnancy or lactation; (5) the history of periodontal treatment during the preceding 6 months; (6) the history of antimicrobial, anti-inflammatory, immunosuppressive, and/or lipid-lowering therapies during the preceding 6 months; (7) regular use of mouth rinses containing antimicrobial drugs; and (8) use of orthodontic appliances. After assessment of the inclusion and exclusion criteria, the height, weight, and waist circumference (WC) were measured for the included patients. BMI was calculated as the weight in kilograms divided by the square of height (kg/m^2^). Patients were considered to have Owt/Ob if their BMI was ≥23 kg/m^2^ and WC was ≥85 cm for men or ≥80 cm for women; they were considered Nwt if their BMI was <23 kg/m^2^ and WC was <85 cm for men or <80 cm for women [21, 22]. Peripheral blood samples after overnight fasting were collected for assessment of baseline blood chemistry in all patients.

### 2.3. Assessment of Periodontal Status

After recruitment, all participants were referred to the Periodontal Clinic, Dental Hospital, Faculty of Dentistry, Mahidol University. They were subjected to a baseline full-mouth periodontal examination by one calibrated periodontist (PL) who had more than 30 years of experience and was unaware of each participant's BMI status. Periodontal measurements were taken at six sites per tooth (i.e., mesiobuccal, midbuccal, distobuccal, mesiolingual, midlingual, and distolingual), using a standard manual probe (PCPUNC 15; Hu-Friedy, Chicago, IL). The following clinical parameters were assessed: the percentage of sites with plaque (i.e., plaque score), the percentage of sites with bleeding on probing (BOP), the probing depth (PD), and the distance from the gingival margin to the cementoenamel junction. In addition, the clinical attachment level (CAL; i.e., the distance from the cementoenamel junction to the most apical portion of the pocket) was calculated as the sum of PD plus the distance from the gingival margin to the cementoenamel junction at each site.

The criteria proposed by the American Academy of Periodontology (with some modifications) were used in this study for diagnosis of periodontitis [23, 24]. Participants with SP were those who had CAL ≥5 mm in ≥30% of sites; at least one of these sites had PD ≥ 4 mm with BOP. Participants who had less severe disease were regarded as participants without SP.

### 2.4. Nonsurgical Periodontal Treatment and Reassessment of Periodontal Status

After baseline periodontal examination and blood sample collection, each participant was subjected to NSPT, which consisted of oral hygiene instruction, scaling, and root planing. A trained periodontist (NV) administered treatment. Oral hygiene instruction was given in accordance with individual needs to achieve a plaque score of <30% after treatment. Scaling and root planing under local anesthesia were performed on a quadrant-by-quadrant basis using a piezoelectric ultrasonic scaler (P5 Newtron, Acteon, Düsseldorf, North Rhine-Westphalia), followed by Gracey curettes (Gracey, Hu-Friedy, Chicago, IL), until calculus could not be detected and the root surface felt smooth. Treatment (2–6 visits) was completed within 2 weeks. No pharmacological interventions were prescribed during treatment. The periodontal statuses of all participants were subsequently reassessed by the calibrated periodontist (PL), and oral hygiene care was provided at 3 and 6 months after NSPT by the trained periodontist (NV) (Supplementary Figure S1).

### 2.5. Assessment of Serum Levels of Leptin, Adiponectin, and CRP

Serum levels of leptin, adiponectin, and CRP were evaluated at baseline, as well as at 3 and 6 months after NSPT. Two milliliters of serum from blood samples were separated by centrifugation and then aliquoted and stored at −80°C. Serum samples were thawed at room temperature, and cytokine measurement was performed by enzyme-linked immunosorbent assay using commercial kits for leptin, adiponectin, and CRP (R&D Systems, Minneapolis, MN). Each serum sample was analyzed in duplicate. All assays were conducted in accordance with the manufacturer's instructions. The absorbance of each well was read using a microplate reader at 450 nm with a 650 nm reference wavelength. A blinded operator (ST) performed the assays.

### 2.6. Statistical Analysis

The sample size to ensure adequate power for this study was calculated considering a difference of ≥0.55 *μ*g/ml of CRP between Owt/Ob and Nwt groups and assuming a standard deviation of 0.50 *μ*g/ml, based on the findings of a previous cross-sectional study performed by our group [14]. Fourteen patients per group in the two main groups (Owt/Ob and Nwt) were judged to be sufficient to provide 80% power with an *α* of 0.05. Therefore, the number of participants for each of the four study groups was set at seven.

The data were analyzed with statistical software (SPSS Statistics for Windows, v.22.0, IBM, Armonk, NY). The normality of the data distribution was assessed using the Shapiro–Wilk test. Cytokine values were normalized using logarithmic transformation. The mean age, FBS level cholesterol level, triglycerides level, CRP level, leptin level, adiponectin level, and periodontal status were compared among groups at baseline by using the Kruskal–Wallis test (comparisons among four groups) and Mann–Whitney *U* test (comparisons between two groups). The percentage distributions of participants with different patient profiles were compared using the chi-squared test. Values of BMI, WC, leptin, adiponectin, and CRP at baseline and after NSPT in each group were compared using the Friedman test, followed by the Wilcoxon matched-pairs signed-rank test. Changes in all four groups were compared between baseline and 3 months or between baseline and 6 months with respect to the plaque score, BOP, CAL, and PD; these comparisons were performed by means of the Kruskal–Wallis test. Regression analyses adjusted for the effects of age, sex, BMI, and WC were further analyzed to explore the associated factors with improvement of PD. The statistical significance threshold was set at *p* < 0.05 for all analyses.

## 3. Results

### 3.1. Participant Characteristics at Baseline

Of the 260 participants screened, 229 were excluded because they did not meet the inclusion criteria; thus, 31 Thai individuals participated in this study. During the follow-up period, one patient in the Nwt with SP group developed tuberculosis, while one patient in the Owt/Ob with SP group was treated with anti-inflammatory drugs. Accordingly, these patients were excluded from the analysis. Therefore, the study participants consisted of 6 patients with Owt/Ob who exhibited SP, 11 patients with Owt/Ob who did not exhibit SP, 5 Nwt patients with SP, and 7 Nwt patients without SP (Supplementary Figure S1).

Twenty-three (79.3%) patients were women. Patients in the Owt/Ob with SP group were significantly older than patients without SP, both in the Owt/Ob (*p* = 0.01) and Nwt groups (*p* = 0.02). Although patients in the Owt/Ob group exhibited higher FBS and triglycerides levels than patients in the Nwt group (*p* = 0.03 and *p* = 0.02, respectively), most blood chemistry values were at normal levels. Cholesterol levels also did not significantly differ among groups (*p* = 0.15) (Table 1).

In all groups, the patients exhibited similar distributions regarding exercise and the education level (*p* = 0.10 and *p* = 0.35, respectively). However, there was a significantly higher number of current alcohol consumers in the Owt/Ob group than in the Nwt group (*p* = 0.02). Regarding brushing habits, all patients brushed their teeth at least twice daily (Table 1).

### 3.2. Periodontal Status at Baseline

At baseline, PD, CAL, and BOP were higher in patients with SP than in patients without SP for both Owt/Ob and Nwt groups (*p* < 0.001, *p* < 0.001, and *p* = 0.03 respectively). However, BOP percentages in the Owt/Ob group did not significantly differ between patients with SP and patients without SP (*p* = 0.73). No statistically significant differences in plaque scores were found among participants in any of the groups (*p* = 0.21) (Table 1).

### 3.3. Cytokine Levels at Baseline

Patients in the Owt/Ob group had significantly higher leptin levels than patients in the Nwt group, irrespective of periodontitis status (*p* < 0.03). However, patients with SP tended to have higher leptin levels than patients without SP (Table 1). In contrast, the mean adiponectin and CRP levels did not significantly differ among the four groups. Notably, CRP levels were higher in patients in the Owt/Ob group than in patients in the Nwt group (Table 1).

### 3.4. Participant Characteristics after Periodontal Treatment

There were no statistically significant differences in BMI and WC among groups after NSPT. Notably, five women in the Nwt without SP group exhibited significantly greater BMI and WC, compared with baseline (*p* = 0.008 and *p* = 0.005, respectively). Moreover, nine of 11 patients in the Owt/Ob without SP group had significantly greater WC (*p* = 0.027).

### 3.5. Periodontal Status after Periodontal Treatment

**Figure 1:**
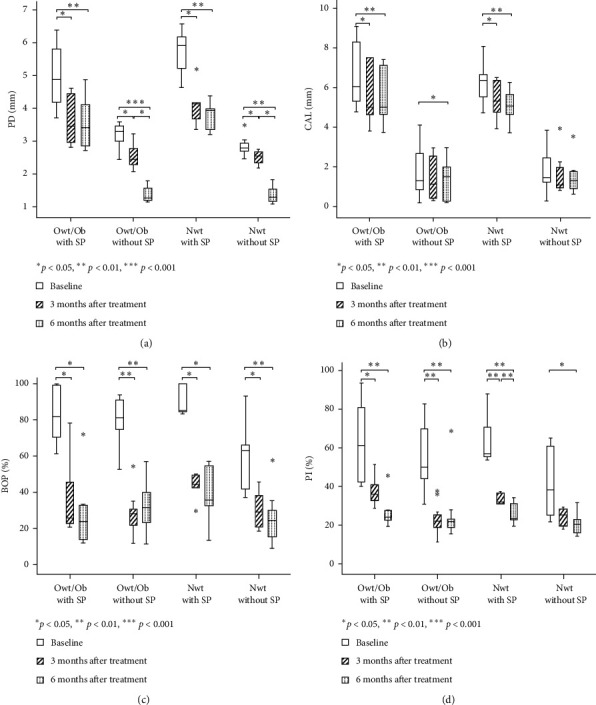
Periodontal parameters and plaque scores at baseline, as well as at 3 and 6 months after NSPT, according to body mass index (BMI) and periodontal disease status: (a) probing depth (PD), (b) clinical attachment level (CAL), (c) bleeding on probing (BOP), and (d) plaque (Pl) score. Differences were compared by the Friedman test; significant differences for pairwise comparisons were determined by the Wilcoxon matched-pairs signed-rank test.

After NSPT, the PD, BOP, and plaque scores of the patients in all four groups exhibited significant reductions at 3 and 6 months (*p* < 0.05) (Figure 1 and Table 2). Except for Nwt patients without SP, no statistically significant changes were found in plaque scores at 3 months after receiving NSPT (Figure 1(d)).

Regarding CAL, patients in the Owt/Ob with SP and Nwt with SP groups had lower CAL at 3 and 6 months after NSPT (*p* < 0.05 and *p* < 0.01, respectively). An opposite trend was observed in patients in the Owt/Ob without SP group at 6 months after NSPT (*p* < 0.05). Furthermore, CAL tended to decrease among patients in the Nwt without SP group at both 3 and 6 months after NSPT, but these differences were not statistically significant (Figure 1(b)).

Among the four groups, changes between baseline and 3 months or baseline and 6 months did not significantly differ for plaque scores, percentage of BOP, or CAL, although the change in PD significantly differed among groups (*p* < 0.001). Adjustment for the effects of age, sex, plaque score, BMI, and WC caused this difference in improvement of PD to disappear during regression analyses (data not shown).

### 3.6. Cytokine Levels after Periodontal Treatment

The median (1^st^ and 3^rd^ quartile) serum levels of leptin, adiponectin, and CRP in all groups at baseline, as well as at 3 and 6 months after NSPT, are shown in Table 3.

### 3.7. Changes in Leptin Levels at 3 and 6 Months after Periodontal Treatment

**Figure 2:**
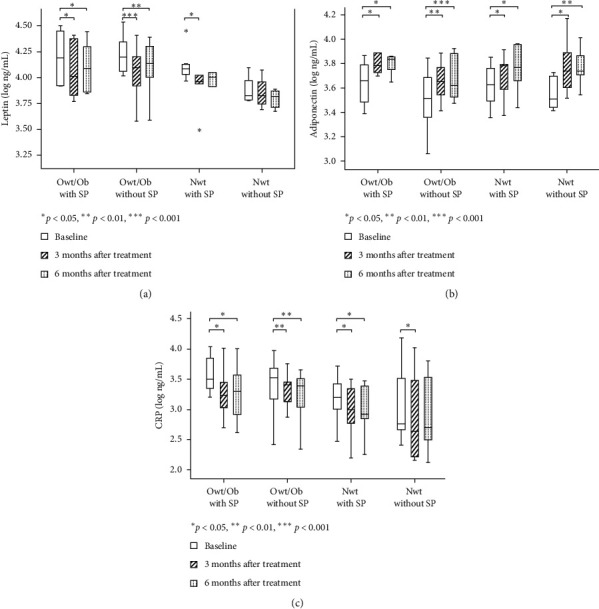
Cytokine levels at baseline, as well as at 3 and 6 months after NSPT, according to body mass index (BMI) and periodontal disease status: (a) leptin, (b) adiponectin, and (c) C-reactive protein. Differences were compared by the Friedman test; significant differences for pairwise comparisons were determined by the Wilcoxon matched-pairs signed-rank test.

Compared with baseline, the mean leptin levels at 3 months after NSPT were significantly lower among patients in the Owt/Ob group, irrespective of periodontitis status, as well as among patients in the Nwt with SP group (*p* = 0.01, *p* < 0.001, and *p* = 0.01, respectively). Compared with baseline, the mean leptin levels at 6 months after NSPT remained significantly lower among patients in the Owt/Ob group, irrespective of periodontitis status (*p* = 0.01 and *p* = 0.009). However, changes in leptin levels among patients in the Nwt without SP group were not significantly different at any timepoints after treatment (Figure 2(a)).

### 3.8. Changes in Adiponectin Levels at 3 and 6 Months after Periodontal Treatment

Compared with baseline, significant increases in adiponectin levels were detected at 3 and 6 months after NSPT in all four groups: Owt/Ob with SP, Owt/Ob without SP, Nwt with SP, and Nwt without SP (*p* = 0.009, *p* < 0.001, *p* = 0.015, and *p* = 0.004, respectively). However, differences between 3 and 6 months were not statistically significant (Figure 2(b)).

### 3.9. Changes in CRP Levels at 3 and 6 Months after Periodontal Treatment

Similar to the patterns observed for leptin levels, the mean CRP levels at 3 months after NSPT, compared with baseline, were significantly lower in all four groups: Owt/Ob with SP, Owt/Ob without SP, Nwt with SP, and Nwt without SP (*p* = 0.03, *p* = 0.001, *p* = 0.03, and *p* = 0.02, respectively). At 6 months after NSPT, the mean CRP levels remained significantly lower than baseline among patients in the following three groups: Owt/Ob with SP, Owt/Ob without SP, and Nwt with SP. Nevertheless, no significant differences in CRP levels were observed between 3 and 6 months in any of the four groups (Figure 2(c)).

## 4. Discussion

To the best of our knowledge, this is the first prospective interventional study to concurrently demonstrate considerable improvement of serum leptin, adiponectin, and CRP levels at 3 and 6 months after NSPT in groups of patients with Owt or Ob, with or without, SP and Nwt patients with or without SP. Although there have been some prospective interventional studies [15–18, 20], participants in the previous reports were typically divided into two groups: Owt/Ob with periodontitis and Nwt with periodontitis. The present study classified participants into four groups, used 3- and 6-month follow-up analyses, and implemented rigorous inclusion criteria. Thus, the present study is more robust in terms of understanding how serum leptin, adiponectin, and CRP are related to SP and Owt/Ob.

In this study, the threshold for Owt (23.00 kg/m^2^) was lower than the threshold used in other studies [15–18, 20]; however, it is relevant for the Thai population [2]. Furthermore, WC ≥ 85 cm in men and WC ≥ 80 cm in women, both of which indicate central obesity and are valid indices of visceral fat accumulation [22], were used in combination with BMI to diagnose patients with Owt or Ob. We strictly use these indices (BMI and WC) for Asian populations, as provided by the World Health Organization; these indices consider population characteristics, environments, nutrition, and socioeconomic factors [21]. Although the present study included a small number of participants, our indices were robust for assessment of central obesity, which influences generalized inflammation in the body, as well as inflammation in the oral cavity [25]. Participants with diabetes mellitus or other health problems that could influence inflammation during the follow-up period were excluded from the study (two patients).

The mean age of participants in the Owt/Ob with SP group was highest among the four groups; this may be related to the presence of chronic periodontal disease in these patients. However, within-group comparisons were implemented to test our hypothesis; therefore, age differences did not bias our overall results. Moreover, participants with SP in this study did not exhibit significant differences in mean PD and percentage of BOP at baseline, irrespective of Nwt or Owt/Ob status. The mean levels of FBS, triglycerides, and cholesterol were also in the normal ranges for all participants. Consequently, these parameters did not affect the overall systemic inflammation or the results of NSPT.

The mean of the deepest PD and the mean of the most severe CAL of each tooth were used in this study. These measurements represent the greatest alterations in the most inflamed areas, which affect systemic inflammation [14], especially in patients with SP. After NSPT, periodontal parameters were improved; PD, CAL, BOP, and plaque scores decreased for participants in all four groups. The results of this study were similar to the findings of previous studies, irrespective of Nwt or Owt/Ob status [16–18, 20]. However, patients in the Nwt without SP group exhibited no changes in CAL or plaque score because of their good periodontal status at baseline.

Furthermore, the mean serum leptin levels at baseline were significantly higher in patients with Owt or Ob than in Nwt patients, irrespective of periodontitis condition. However, the mean levels of adiponectin and CRP did not significantly differ among the four groups. These findings are consistent with the results of previous studies by Altay et al. [16] and Gonçalves et al. [20]. A possible explanation is that patients with Ob have leptin resistance, a feature that contributes to the pathology of obesity. Leptin resistance and reduced leptin sensitivity lead to higher leptin levels in patients with Ob than in Nwt patients [4].

Notably, the levels of serum leptin were significantly reduced at 3 and 6 months after NSPT, especially in patients with Owt or Ob, irrespective of periodontitis status. This result is consistent with the previous findings reported by Altay et al. [16]. Moreover, we found that the serum levels of leptin were significantly reduced in Nwt patients with SP at 3 months after NSPT. Shimada et al. reported similar results, although participants in their study were not classified by weight status [26]. However, Gonçalves et al. did not observe altered leptin levels at 1 year after NSPT [20]. Evidence thus far is contradictory regarding the overall benefits of NSPT on leptin levels. Possible explanations for differences among studies may be related to their inclusion/exclusion criteria and parameters used to define obesity, sample sizes, methods of adipokine detection, severities of periodontitis and obesity, and interpatient variabilities in the systemic response to periodontal therapy [27].

In all four study groups, the levels of adiponectin were significantly elevated at 3 and 6 months after NSPT, both among participants with SP, irrespective of Owt/Ob status, and among participants with Nwt or Owt/Ob, irrespective of periodontitis status. This result supports our hypothesis because periodontitis and Owt/Ob status independently reduce adiponectin levels [14]. The reduction of local inflammation in the oral cavity by NSPT thus could enhance serum adiponectin levels. However, conflicting results have been reported elsewhere [20]. The variation of periodontal statuses among study participants may have influenced the small change in adiponectin levels observed by Gonçalves et al. [20].

The association between periodontitis and an increase of serum CRP has been reported elsewhere [13]. Our study showed that the mean CRP levels at 3 and 6 months after NSPT were reduced in all four groups, except at 6 months after NSPT in Nwt patients without SP. This finding is consistent with the previous report by Al-Zahrani and Alghamdi [18]. However, their study only included female patients for 2 months of follow-up; moreover, it used only BMI to determine whether patients had Ob [18]. In contrast, the study by Altay et al. did not demonstrate altered CRP levels at 3 months after treatment [16]. These discrepancies might be related to the criteria used to define Ob and periodontitis. In the study by Altay et al., the criteria were less specific, and the oral cavity had less generalized inflammation, compared with the current study [16].

In this study, BMI and WC both increased in Nwt patients without SP. Patients in the Owt/Ob without SP group also exhibited significantly greater WC over time. These results show that, despite elevated BMI and WC, CRP levels decreased in Nwt patients without SP, whereas adiponectin levels increased at 3 months after NSPT. Similarly, levels of leptin and CRP were both reduced at 3 and 6 months after NSPT, whereas levels of adiponectin were elevated, despite the undesirable change in WC among patients in the Owt/Ob without SP group. WC and BMI were not associated with improvement in PD, based on regression analyses in our study. The evidence from a meta-analysis regarding the effect of NSPT on periodontal clinical response, in which PD was used as a surrogate parameter for periodontal healing between patients with Ob and those with Nwt, remains controversial [10, 28]. Bartold suggested that obesity must be a factor into a personalized care model for managing periodontal disease [29]. Nevertheless, the present results support the findings in our previous cross-sectional study in a Thai population: periodontitis and Owt or Ob status independently cause changes in plasma levels of inflammatory mediators, adiponectin, and CRP [14].

Regarding the risks of cardiovascular disease, especially coronary heart disease, CRP levels >3 *μ*g/ml before periodontal treatment are suggestive of a high risk of coronary heart disease [30]. Patients with coronary heart disease with or without periodontitis presented higher levels of CRP than those of healthy individuals [31, 32]. Although patients exhibited inconsistent BMI and WC during periodic recall, CRP levels were reduced to low risk (<1.0 *μ*g/ml) or intermediate risk (1.0–3.0 *μ*g/ml). Furthermore, levels of adiponectin, which is an anti-inflammatory mediator, were also elevated after NSPT in our study. These results confirm the systemic effects of NSPT on the systemic reduction of serum inflammatory mediators due to periodontal disease, irrespective of BMI or WC, and probably on decreased risk of coronary heart disease.

This preliminary study had some limitations. The number of participants was limited due to the use of rigorous inclusion criteria, which were implemented to minimize the effects of confounding factors. Selection bias may have been involved because the included participants were patients who visited our hospital; there may also have been some biological variation among the patients. Therefore, these results should be carefully interpreted if they are generalized to other groups. More representative sample populations are needed in future investigations. Long-term longitudinal studies are needed to confirm the importance of NSPT in improvement of inflammatory mediators and reduction of the risk of cardiovascular disease. Despite these limitations, the findings of this study show that conservative periodontal treatment (i.e., scaling and root planing) can considerably reduce serum levels of leptin and CRP; it can also enhance serum levels of adiponectin during the 3–6-month recall period, especially in patients with Owt or Ob, irrespective of periodontitis severity.

## 5. Conclusion

NSPT reduces serum levels of CRP and leptin while enhancing serum levels of adiponectin, in Thai patients with Owt or Ob, irrespective of periodontitis severity. These results support the importance of dental professionals in reduction of systemic inflammatory mediators in patients with a common noncommunicable disease.

## Figures and Tables

**Table 1:** Characteristics of participants, according to BMI and periodontal disease status at baseline.

	Owt/Ob			Nwt			*p* ^‡^
	With SP	Without SP	*p* ^ϯ^	With SP	Without SP	*p* ^ϯ^	
Age (years)	52.50 (51.50, 65.50)	46.00 (39.00, 50.00)	0.01	46.00 (41.50, 53.00)	43.00 (36.00, 48.00)	0.27	0.02
Sex							
Male	1	3	—	1	1	—	—
Female	5	8		4	6		
BMI (kg/m^2^)	25.07 (23.96, 29.20)	25.00 (24.16, 32.18)	—	21.70 (19.28, 22.20)	19.98 (18.14, 22.32)	—	—
Waist circumference (cm)							
Male	97.00	92.00 (91.00, —)	—	83.00	80 .00	—	—
Female	90.00 (81.50, 92.25)	84.00 (81.25, 89.88)		74.00 (67.75, 78.75)	73.00 (66.75, 67.75)		
Blood chemistry							
Fasting blood sugar (mg/dl)	99.50 (94.50, 114.25)	95.00 (87.00, 108.00)	0.22	91.00 (83.50, 96.00)	86.00 (82.00, 91.00)	0.43	0.03
Cholesterol (mg/dl)	217.00 (203.50, 229.00)	228.00 (198.00, 235.00)	0.88	217.00 (167.50, 251.50)	181.00 (158.00, 216.00	0.34	0.15
Triglycerides (mg/dl)	86 (70.50, 190.25)	148.00 (111.00, 218.00)	0.12	88.00 (65.50, 115.50)	82.00 (45.00, 100.00)	0.43	0.02
Personal history							
Exercise							
Yes (≥3 times/week)	2 (33.30%)	3 (27.30%)	0.09	2 (40.00%)	3 (42.90%)	0.56	0.10
No (<3 times/week)	4 (66.70%)	8 (72.70%)		3 (60.00%)	4 (57.10%)		
Education							
<Bachelor's degree	4 (66.70%)	4 (63.60%)	0.81	3 (60.00%)	0 (0.00%)	0.08	0.35
≥Bachelor's degree	2 (33.30%)	7 (36.40%)		2 (40.00%)	7 (100.00%)		
Alcohol consumption							
Current	1 (16.70%)	5 (45.50%)	0.23	2 (40.00%)	0 (0.0%)	0.02	0.02
Never/former	5 (83.30%)	6 (54.50%)		3 (60.00%)	7 (100.00%)		
Brushing habit							
≤1 time/day	0 (0.00%)	0 (0.00%)	—	0 (0.00%)	0 (0.00%)	—	—
≥2 times/day	6 (100.00%)	11 (100.00%)		5 (100.00%)	7 (100.00%)		
Periodontal parameters							
PD (mm)	3.56 (2.92, 4.30)	2.43 (2.22, 2.61)	<0.001	3.95 (3.35, 4.38)	2.13 (1.91, 2.26)	0.003	<0.001
CAL (mm)	4.19 (3.73, 6.42)	0.55 (0.22, 1.73)	<0.001	4.36 (3.27, 5.17)	0.74 (0.36, 1.54)	0.005	<0.001
BOP (%)	81.64 (68.24, 99.31)	81.25 (74.40, 91.23)	0.73	85.33 (83.95, 100.00)	62.96 (36.90, 66.67)	0.02	0.03
Plaque score (%)	60.94 (41.61, 83.96)	50.00 (41.67, 72.62)	0.59	56.82 (54.48, 79.10)	38.10 (23.81, 64.81)	0.15	0.21
Cytokine levels							
Leptin (ng/mL)	16.17 (8.32, 28.93)	15.76 (11.40, 23.35)	1.00	12.13 (9.93, 20.70)	6.69 (5.99, 11.29)	0.05	0.03
Adiponectin (*μ*g/mL)	4.57 (2.90, 6.45)	3.29 (2.00, 4.82)	0.30	4.23 (2.69, 6.44)	3.22 (2.70, 5.02)	0.64	0.64
CRP (*μ*g/mL)	3.17 (2.08, 8.04)	3.35 (1.41, 5.64)	0.59	1.58 (0.66, 3.97)	0.58 (0.41, 4.36)	0.76	0.34

Data are shown as median (*Q*_1_ and *Q*_3_) or *n* (%). ^Ϯ^Mann–Whitney *U* test or chi-square. ^‡^Kruskal–Wallis test or chi-square. PD, probing depth (mean of probing depth of full mouth); CAL, clinical attachment level (mean of the clinical attachment level of full mouth); BOP, bleeding on probing; BMI, body mass index; CRP, C-reactive protein; Owt, overweight; Ob, obesity; Nwt, normal weight; SP, severe periodontitis.

**Table 2:** Periodontal parameters at baseline, as well as at 3 and 6 months after periodontal treatment, according to BMI and periodontal disease status.

	Owt/Ob		Nwt	
	With SP	Without SP	With SP	Without SP
PD (mm)				
Baseline	4.88 (4.07, 5.96)	3.29 (2.85, 3.50)	5.91 (4.93, 6.38)	2.79 (2.63, 3.04)
3 months	3.50 (2.90, 4.48)	2.44 (2.22, 2.92)	4.14 (3.52, 4.66)	2.54 (2.31, 2.70)
6 months	3.41 (2.81, 4.30)	1.26 (1.19, 1.67)	3.96 (3.28, 4.17)	1.29 (1.11, 1.70)
*p*^†^	0.006	<0.001	0.015	0.002
CAL (mm)				
Baseline	6.04 (5.16, 8.48)	1.30 (0.67, 3.00)	6.36 (5.12, 7.37)	1.46 (1.18, 2.96)
3 months	5.02 (4.43, 7.50)	1.57 (0.39, 2.72)	5.29 (4.33, 6.43)	1.08 (0.92, 2.25)
6 months	5.02 (4.42, 7.19)	1.50 (0.26, 2.39)	5.05 (4.18, 5.96)	1.33 (0.89, 1.79)
*p*^†^	0.004	0.022	0.007	0.317
BOP (%)				
Baseline	81.64 (68.24, 99.31)	81.25 (74.40, 91.23)	85.33 (83.95, 100.00)	62.96 (36.90, 66.67)
3 months	25.25 (22.04, 52.66)	28.47 (21.60, 30.36)	43.83 (36.20, 49.67)	29.17 (19.64, 41.36)
6 months	23.96 (13.30, 40.51)	31.48 (20.37, 44.64)	35.80 (22.95, 55.84)	24.31 (11.90, 35.19)
*p*^†^	0.009	<0.001	0.022	0.002
Plaque score (%)				
Baseline	60.94 (41.61, 83.96)	50.00 (41.67, 72.62)	56.82 (54.48, 79.10)	38.10 (23.81, 64.81)
3 months	35.63 (31.60, 43.40)	22.22 (17.59, 26.54)	32.72 (31.25, 36.81)	25.64 (18.52, 29.17)
6 months	25.37 (23.07, 33.08)	21.43 (17.90, 23.21)	23.46 (21.03, 32.59)	20.24 (14.88, 24.07)
*p*^†^	0.006	0.001	0.007	0.028

Data are shown as median (Q1, Q3). ^†^, Friedman test; PD, probing depth (mean of deepest probing depth of each tooth); CAL, clinical attachment level (mean of the most severe clinical attachment level of each tooth); Owt, overweight; Ob, obesity; Nwt, normal weight; SP, severe periodontitis.

**Table 3:** Levels of cytokines at baseline, as well as at 3 and 6 months after periodontal treatment, according to BMI and periodontal disease status.

	Owt/Ob		Nwt	
	With SP	Without SP	With SP	Without SP
Leptin (ng/mL)				
Baseline	16.17 (8.32, 28.93)	15.76 (11.40, 23.35)	12.13 (9.93, 20.70)	6.69 (5.99, 11.29)
3 months	10.33 (6.55, 24.21)	12.63 (7.06, 18.02)	9.04 (5.92, 16.06)	6.70 (4.91, 10.83)
6 months	12.80 (7.18, 21.81)	13.61 (9.90, 20.42)	10.10 (6.16, 17.25)	6.70 (4.72, 7.75)
*p*^†^	0.009	<0.001	0.015	0.156
Adiponectin (*μ*g/mL)				
Baseline	4.57 (2.90, 6.45)	3.29 (2.00, 4.82)	4.23 (2.69, 6.44)	3.22 (2.70, 5.02)
3 months	6.09 (5.24, 7.71)	4.36 (3.31, 6.22)	6.04 (3.12, 7.19)	5.54 (3.31, 8.34)
6 months	6.81 (5.35, 7.96)	4.18 (3.25, 7.68)	5.85 (3.65, 9.08)	5.48 (4.86, 7.92)
*p*^†^	0.009	<0.001	0.015	0.004
CRP (*μ*g/mL)				
Baseline	3.17 (2.08, 8.04)	3.35 (1.41, 5.64)	1.58 (0.66, 3.97)	0.58 (0.41, 4.36)
3 months	1.88 (0.93, 4.68)	2.59 (1.26, 2.96)	0.98 (0.37, 2.69)	0.43 (0.15, 4.25)
6 months	2.10 (0.72, 5.29)	2.47 (0.88, 3.26)	0.84 (0.44, 2.71)	0.50 (0.20, 6.33)
*p*^†^	0.011	<0.001	0.022	0.018

Data are shown as median (Q1, Q3). ^†^, Friedman test; CRP, C-reactive protein; Owt, overweight; Ob, obesity; Nwt, normal weight; SP, severe periodontitis.

## Data Availability

Access to data is restricted due to legal and ethical concerns. Nevertheless, data used to support the findings of this study are available from the corresponding author upon request.

## Supplementary Materials

Supplementary Materials Flowchart of the study protocol: PD, probing depth; CAL, clinical attachment level; BOP, bleeding on probing; BMI, body mass index; WC, waist circumference; Owt/Ob, patients with overweight or obesity; Nwt, normal weight patients; SP, severe periodontitis.
