# Supplementary material for: Periodontal Treatment Improves Serum Levels of Leptin, Adiponectin, and C-Reactive Protein in Thai Patients with Overweight or Obesity
Source: Int J Dent. 2021 Feb 2;2021:6660097. doi: 10.1155/2021/6660097 (PMC7872757; doi:10.1155/2021/6660097)
Supplement: Supplementary Materials — Flowchart of the study protocol: PD, probing depth; CAL, clinical attachment level; BOP, bleeding on probing; BMI, body mass index; WC, waist circumference; Owt/Ob, patients with overweight or obesity; Nwt, normal weight patients; SP, severe periodontitis. [file 6660097.f1.docx]

Screening

(interview, review of medical history, assessment of number of teeth, BMI, WC)

Participating patients (n=29)

Owt/Ob

with SP

(n=6)

Baseline

3-month follow-up

Blood

6-month follow-up

Blood examination (level of C-reactive protein, leptin, and adiponectin)

Full mouth scaling, root planing, and polishing

Plaque score ≤30%

Blood examination (levels of C-reactive protein, leptin, and adiponectin)

Periodontal examination (PD, CAL, BOP, Plaque score), BMI, WC

Blood examination (levels of C-reactive protein, leptin, and adiponectin)

Periodontal examination (PD, CAL, BOP, Plaque score), BMI, WC

Patients attending General/Special clinic,

Tropical Medicine Hospital

(n=260)

Full-mouth periodontal examination (PD, CAL, BOP, Plaque score)

Baseline

Nwt

with SP

(n=5)

Nwt

without SP

(n=7)

Owt/Ob

without SP

(n=11)

**Flowchart of the study protocol**

PD = probing depth

CAL = clinical attachment level

BOP = bleeding on probing

BMI = body mass index

WC = waist circumference

Owt/Ob = patients with overweight or obesity

Nwt = normal-weight patients

SP = severe periodontitis
